# Curcumin Inhibits Papillary Thyroid Cancer Cell Proliferation by Regulating lncRNA LINC00691

**DOI:** 10.1155/2022/5946670

**Published:** 2022-02-26

**Authors:** Zhihua Li, Youbing Gao, Lijun Li, Shanshan Xie

**Affiliations:** ^1^Department of Thyroid and Breast Surgery, Hubei Provincial Hospital of Traditional Chinese Medicine, Wuhan 430061, China; ^2^Hubei Academy of Traditional Chinese Medicine, Wuhan 430074, China

## Abstract

Papillary thyroid cancer (PTC) is a type of epithelial-derived differentiated TC that reportedly accounts for a majority of TCs. Curcumin, a polyphenolic compound and a member of the Zingiberaceae (ginger) family derived from turmeric plants, can exhibit anticancer effects. Herein, we aimed to investigate the effect of curcumin on PTC and elucidate underlying mechanisms. Accordingly, PTC B-CPAP cells were treated with curcumin, in combination with/without long noncoding RNA LINC00691 inhibition, to determine the effect of curcumin and its relationship with LINC00691 in PTC cells. We observed that curcumin treatment decreased B-CPAP cell proliferation and promoted apoptosis. Curcumin inhibited LINC00691 expression in B-CPAP cells. Curcumin administration or si-LINC00691 transfection alone promoted ATP levels, inhibited glucose uptake and lactic acid levels, and inhibited lactate dehydrogenase A and hexokinase 2 protein expression in B-CPAP cells, which were further enhanced by combination treatment. Moreover, curcumin administration or si-LINC00691 transfection alone inhibited p-Akt activity, further suppressed by combination treatment. Akt inhibition promoted apoptosis and suppressed the Warburg effect in B-CPAP cells. In conclusion, our findings indicate that curcumin promotes apoptosis and suppresses proliferation and the Warburg effect by inhibiting LINC00691 in B-CPAP cells. The precise molecular mechanism might be mediated through the Akt signaling pathway, providing a theoretical basis for the treatment of PTC with curcumin.

## 1. Introduction

Current strategies to treat cancer remain limited. Thyroid cancer (TC) is a prevalent endocrine malignancy worldwide, accounting for approximately 300,000 new cases in 2012 and 40,000 deaths [1–3]. TC is commonly classified into anaplastic TC, medullary TC, and differentiated TC [4]. Papillary TC (PTC) is an epithelial-derived differentiated TC that accounts for most TC cases [3]. Surgery, radioactive iodine treatment, and chemotherapy are primary options for treating PTC. However, these strategies minimally impact the prognosis of recurrent PTC [5]. Thus, a better understanding of underlying molecular mechanisms and identification of novel agents remains crucial to improve therapeutic strategies for patients with PTC. The use of dietary phytochemicals during the early stages of cancer development could be considered an ideal strategy for cancer prevention. Ginger extract reportedly promotes apoptosis, inhibits cell proliferation, and prevents oxidative stress in liver cancer cells [6]. Two promising peptide sequences of pepsin, present in anticancer [7] phytochemicals, were shown to mitigate cancer progression by inducing apoptosis or intracellular oxidative stress [8]. El-Dakhly et al. have shown that combining aescin and diosmin affords a robust hepatoprotective effect [9], and Amin et al. have found that saffron and its main compounds saffron aldehyde and saffronin regulate the cell cycle and DNA damage repair system [10]. Therefore, comprehensively understanding molecular mechanisms and identifying novel therapeutics are essential to improve treatment strategies for patients with cancer.

Long noncoding RNAs (lncRNAs) are noncoding transcripts that are more than 200 nucleotides long and participate in chromosome X inactivation, maintenance of pluripotency, genomic imprinting, various cellular processes, and the formation of different organs by modulating chromatin, transcription, and translation [11, 12]. Accumulating evidence has demonstrated that lncRNAs play a markedly diverse role in the process of tumor occurrence and development by sponging microRNAs (miRNAs), participating in the regulation of cell proliferation, angiogenesis, migration, and apoptosis [13–16]. Zhou et al. have reported that lncRNA ABHD11-AS1 plays a role in regulating PTC progression through the miR-199a-5p/SLC1A5 axis [16]. In addition, Zhang et al. have suggested that lncRNA NEAT1 regulates PTC progression by regulating miR-129-5p expression [17]. Previous studies have demonstrated the upregulation of miR-451a in PTC, which could be characterized as a potential biomarker for PTC [18, 19]. Using bioinformatic analysis, we noted an interactive relationship between miR-451a and LINC00691. However, the effect of LINC00691 on PTC has not been comprehensively explored.

Numerous natural products are employed to manufacture therapeutic nanomedicines, such as albumin, dendrimers, liposomes, micelles, ceramics, and metal nanoparticles, which can be employed as drug delivery agents and exhibit potential in cancer therapy [20]. One study attempted to develop an effective treatment for liver cancer using magnetite nanoparticles (MNPs) coated with saffronin, the main active ingredient in saffron [21]. In addition, several studies have identified the role of certain natural products in treating and preventing various diseases. For example, camel whey protein hydrolysate, produced by gastric and pancreatic enzymes, acts as an antidiabetic and anti-inflammatory agent [22], and vitamin C and vitamin E can improve metabolic biochemical parameters in diabetic rats [23, 24]. Consequently, research in this field has gained considerable momentum. Curcumin is a polyphenolic compound and a member of the Zingiberaceae (ginger) family derived from turmeric plants [25]. It has been extensively used in Chinese medicine to treat various diseases, including inflammation and cancer [26, 27]. Curcumin was found to participate in the process of pancreatic cancer [28], colorectal cancer [29], and hepatocellular cancer [30] by regulating specific lncRNAs and miRNAs. In addition, curcumin can suppress PTC cell metastasis by downregulating the TGF-beta/Smad2/3 signaling pathway [31]. Curcumin was shown to enhance the anticancer activity of cisplatin in PTC cells and cancer stem-like cells by regulating the JAK/STAT3 signaling [32]. However, the effect of curcumin on PTC and its underlying mechanisms needs to be clarified. We have previously reported that curcumin can inhibit proliferation and invasion by regulating miR-451a (data not shown). However, whether the inhibitory effect of curcumin on PTC is associated with LINC00691 needs to be further elucidated.

In the present study, we detected the relative expression of LINC00691 in PTC cells. Subsequently, PTC B-CPAP cells were treated with curcumin, combined with or without LINC00691 inhibition, to detect the effect of curcumin and its relationship with LINC00691 in PTC cells.

## 2. Materials and Methods

### 2.1. Cell Culture

PTC cell lines B-CPAP, BHT-101, and KTC-1 were supplied by the Shanghai Institutes for Biological Sciences, Chinese Academy of Sciences, and cultured in RPMI-1640 (Hyclone) supplemented with 10% fetal bovine serum (FBS; Gibco), Dulbecco's modified Eagle medium (Hyclone) supplemented with 20% FBS, and RPMI-1640 supplemented with 10% FBS, 1% nonessential amino acids (Invitrogen), 1% Glutamax (Invitrogen), and 1% sodium pyruvate (Sigma), respectively. Thyroid epithelial cells were cultured in RPMI-1640 supplemented with 10% FBS and 1% Glutamax. All cells were maintained at 37°C with 5% CO_2_. The cells were harvested after reaching 80-90% confluency, and LINC00691 expression was detected using quantitative reverse transcription-polymerase chain reaction (qRT-PCR).

### 2.2. qRT-PCR

LINC00691 expression in small interfering- (si-) LINC00691 transfected to different curcumin concentration (2.5, 5, 10, and 20 *μ*M)-treated B-CPAP cells was detected using qRT-PCR. The different concentrations were based on the results from previous studies [33–35]. Whole RNA was extracted from B-CPAP cells using TRIzol (Ambion) and reverse-transfected into cDNA. Subsequently, cDNA was amplified under the following thermocycling conditions: 95°C for 3 min; 40 cycles of denaturation at 95°C for 5 s, annealing at 56°C for 10 s, and extension at 72°C for 25 s; final extension was performed at the rate of 5°C/5 s from 65°C to 95°C. The primary sequences were as follows: LINC00691: forward, 5′-GATGAGAAACGGAAACCC C-3′, reverse, 5′-TGACAGGACGCCGACATT-3′ and GAPDH (internal reference): forward, 5′-CCACTCCTCCACCTTTG-3′, reverse, 5′-CCACTCCTCCACCTTTG-3′. The 2^-*ΔΔ*Ct^ method was used to calculate the relative lncRNA expression levels [36].

### 2.3. Cell Counting Kit-8

The cell counting kit-8 (CCK-8) assay was performed to detect B-CPAP cell viability after si-LINC00691 transfection or treatment with different curcumin concentrations. Briefly, 3 × 10^3^ cells in each group were seeded into 96-well plates (100 *μ*L per well) and maintained at 37°C with 5% CO_2_ overnight. After transfection or curcumin treatment for 24 h, the cells were cultured for 4 h with the addition of 10 *μ*L CCK-8 solution (Solarbio) and subjected to a microplate reader (Allsheng) to detect absorbance at 450 nm.

### 2.4. Flow Cytometry

Briefly, 1 × 10^6^ resuspended cells in each group were centrifuged for 5 min at 400 × *g* and 4°C (repeated twice). Next, the cells were resuspended in 200 *μ*L PBS and stained for 30 min with 10 *μ*L Annexin V-fluorescein isothiocyanate (FITC, BD) and 10 *μ*L propidium iodide (PI, BD) in the dark at 4°C. After adding 300 *μ*L PBS, the cells were subjected to flow cytometry (ACEA Biosciences).

### 2.5. Western Blot

Total proteins were extracted from B-CPAP cells using radioimmunoprecipitation assay and quantified using a bicinchoninic acid assay kit (Solarbio). In brief, 20 *μ*g of harvested proteins from each group was separated by 12% sodium dodecyl sulfate-polyacrylamide gel electrophoresis and transferred onto polyvinylidene fluoride membranes. The membranes were then blocked with 5% skim milk at 4°C overnight and cultured for 1 h with primary antibodies against B cell lymphoma-2 (Bcl-2), Bcl-2/Bcl-XL-associated death promoter (Bad), lactate dehydrogenase A (LDHA), hexokinase 2 (HK2), Akt (all supplied by Bioswamp), cleaved caspase-3, phosphorylated (p)-Akt, and GAPDH (an endogenous control) (supplied by Abcam). Subsequently, the membranes were incubated with goat anti-rabbit IgG secondary antibody for 1 h.

### 2.6. Biochemical Assay

Levels of adenosine triphosphate (ATP) (A095) and lactic acid (A019-02), as well as glucose uptake (cat. KA4086) in B-CPAP cells, were evaluated using corresponding commercial kits. The ATP and lactic acid assay kits were supplied by the Nanjing Jiancheng Bioengineering Institute. The glucose uptake assay kit was supplied by Abnova.

### 2.7. Statistical Analysis

Data are presented as the mean ± standard deviation (SD). Statistical differences were analyzed using one-way analysis of variance, followed by Tukey's test. Statistical significance was set at *p* < 0.05.

## 3. Results

### 3.1. LINC00691 Was Overexpressed in B-CPAP Cells and LINC00691 Inhibition Attenuated B-CPAP Cell Proliferation

**Figure 1:**
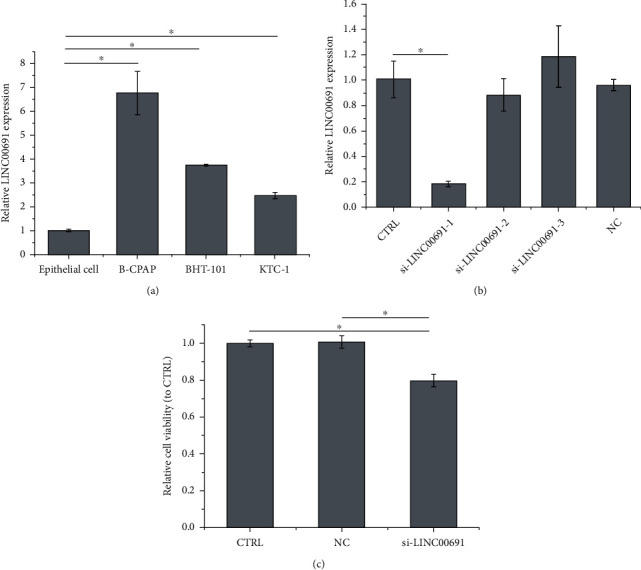
LINC00691 is overexpressed in B-CPAP cells, and LINC00691 inhibition attenuates B-CPAP cell proliferation. (a) qRT-PCR was performed to detect the expression of LINC00691 in PTC cells. (b) qRT-PCR was performed to evaluate the transfection efficiency in B-CPAP cells. (c) CCK-8 assay was performed to examine cell viability. Data are presented as the mean ± standard deviation (SD), *n* = 3, ^∗^*p* < 0.05. CTRL: control; NC: negative control of si-LINC00691; qRT-PCR: quantitative reverse transcription-polymerase chain reaction; PTC: papillary thyroid cancer.

We examined the expression of LINC00691 in B-CPAP, BHT-101, and KTC-1 cells. Compared with thyroid epithelial cells, B-CPAP, BHT-101, and KTC-1 cells showed increased LINC00691 expression. B-CPAP cells were chosen for subsequent investigations owing to the high LINC00691 expression (Figure 1(a)). LINC00691 expression in B-CPAP cells was then inhibited by si-LINC00691 transfection (si-LINC00691-1 was selected for subsequent experiments) (Figure 1(b)). The CCK-8 assay revealed that si-LINC00691 transfection suppressed the viability of B-CPAP cells.

### 3.2. Curcumin Attenuated Proliferation and LINC00691 Expression in B-CPAP Cells

**Figure 2:**
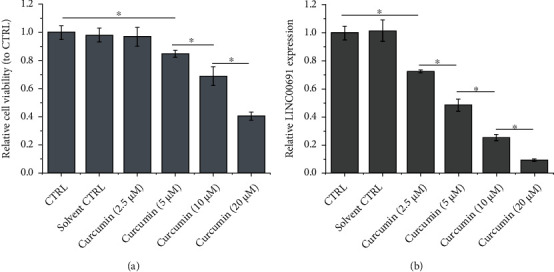
Curcumin attenuates proliferation and LINC00691 expression in B-CPAP cells. (a) CCK-8 assay was performed to detect B-CPAP cell viability. (b) qRT-PCR was performed to detect the expression of LINC00691 in B-CPAP cells. Data are presented as the mean ± standard deviation (SD), *n* = 3, ^∗^*p* < 0.05. CTRL: control; qRT-PCR: quantitative reverse transcription-polymerase chain reaction.

As shown in Figure 2(a), curcumin administration inhibited B-CPAP cell viability in a concentration-dependent manner. The IC_50_ of curcumin in B-CPAP cells was approximately 16 *μ*M, which was selected for subsequent experiments. In addition, curcumin suppressed LINC00691 expression in a concentration-dependent manner (Figure 2(b)).

### 3.3. Curcumin Attenuated Apoptosis by Regulating LINC00691 Expression in B-CPAP Cells

**Figure 3:**
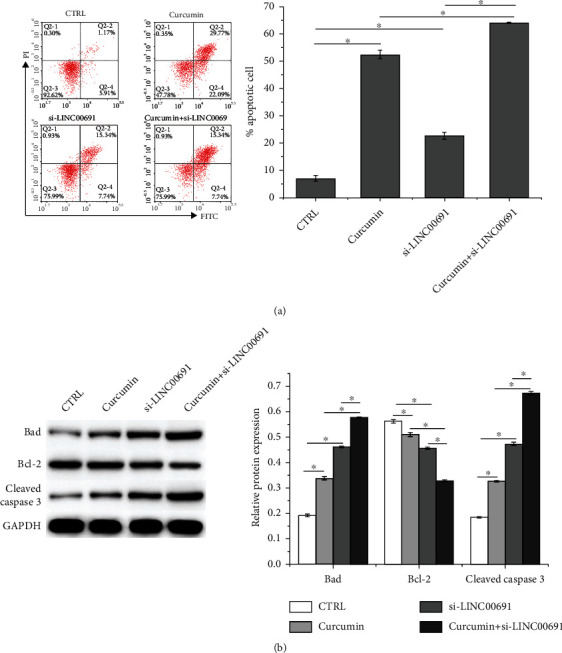
Curcumin attenuates apoptosis by regulating LINC00691 expression in B-CPAP cells. (a) Flow cytometry was performed to detect apoptosis in B-CPAP cells. (b) Western blot was performed to detect the expression of apoptosis-related proteins in B-CPAP cells. Data are presented as the mean ± standard deviation (SD), *n* = 3, ^∗^*p* < 0.05. CTRL: control.

Based on flow cytometric analysis, curcumin administration or si-LINC00691 transfection alone promoted apoptosis in B-CPAP cells, which was further enhanced following combination treatment (Figure 3(a)). Western blot assay results revealed that curcumin administration or si-LINC00691 transfection alone enhanced Bad and cleaved caspase-3 expression and suppressed Bcl-2 expression; combination treatment further strengthened these phenomena (Figure 3(b)).

### 3.4. Curcumin Inhibited the Warburg Effect by Regulating LINC00691 Expression in B-CPAP Cells

**Figure 4:**
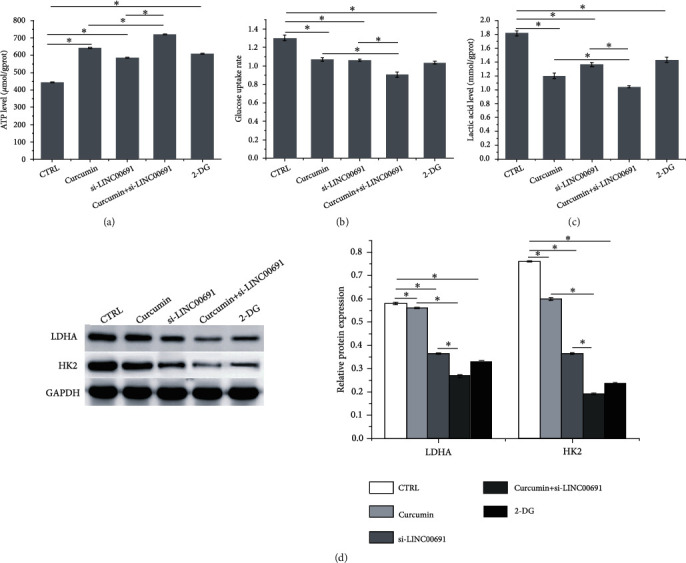
Curcumin inhibits the Warburg effect by regulating LINC00691 expression in B-CPAP cells. The (a) ATP, (b) glucose, and (c) lactic acid levels in B-CPAP cells. (d) Western blot was performed to detect the expression of enzymes involved in the Warburg effect. Data are presented as the mean ± standard deviation (SD), *n* = 3, ^∗^*p* < 0.05. CTRL: control; 2-DG: 2-deoxy-D-glucose.

As shown in Figures 4(a)–4(c), curcumin administration or si-LINC00691 transfection alone enhanced ATP levels and inhibited glucose uptake and lactic acid levels in B-CPAP cells; combination treatment further strengthened these effects. In addition, curcumin administration or si-LINC00691 transfection alone inhibited LDHA and HK2 protein expressions, which were further inhibited following combination treatment with curcumin administration and si-LINC00691 transfection (Figure 4(d)). The effect of curcumin treatment with/without si-LINC00691 transfection was similar to that of 2-deoxy-D-glucose (2-DG, 10 mM), a glucose inhibitor.

### 3.5. Curcumin Attenuated Apoptosis and the Warburg Effect by Regulating the Akt Signaling in B-CPAP Cells

**Figure 5:**
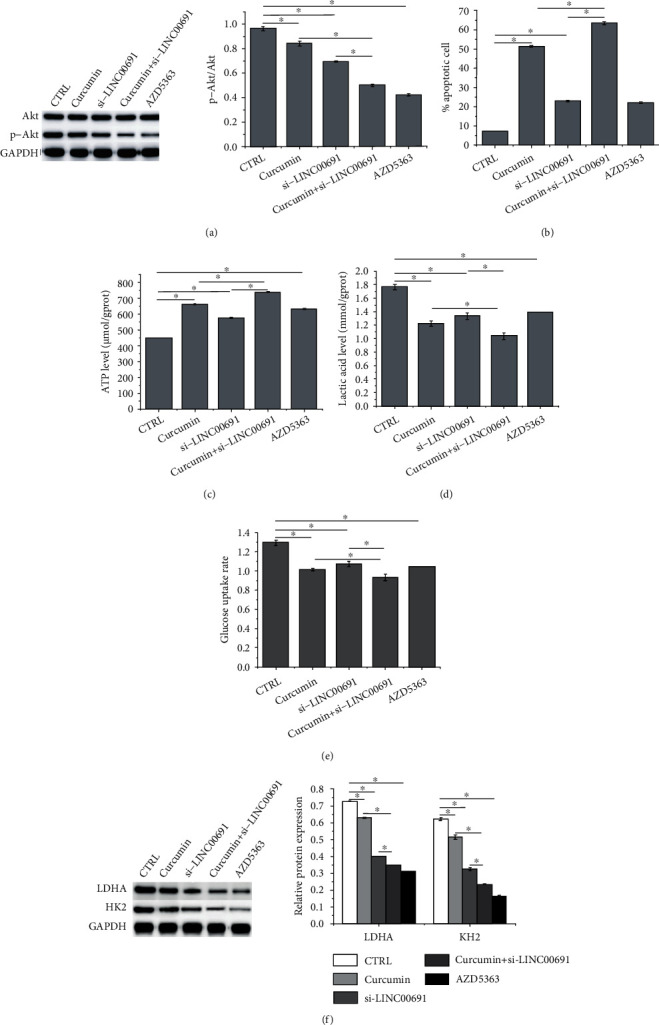
Curcumin attenuates apoptosis and the Warburg effect by regulating the Akt signaling in B-CPAP cells. (a) Western blotting was performed to detect the expression of Akt and p-Akt in B-CPAP cells. (b) Flow cytometry was performed to examine apoptosis in B-CPAP cells. The (c) ATP, (d) glucose, and (e) lactic acid levels in B-CPAP cells. (f) Western blot was performed to detect the expression of enzymes involved in the Warburg effect. Data are presented as the mean ± standard deviation (SD), *n* = 3, ^∗^*p* < 0.05. CTRL: control; AZD: Akt inhibitor.

Based on western blotting analysis, curcumin administration or si-LINC00691 transfection alone inhibited p-Akt activity, which was further inhibited following combination treatment (Figure 5(a)). Furthermore, Akt inhibitor, AZD5363, enhanced apoptosis (Figure 5(b)) and suppressed the Warburg effect in B-CPAP cells, as indicated by increased ATP levels (Figure 5(c)) and decreased lactic acid (Figure 5(d)), glucose uptake (Figure 5(e)), and LDHA and HK2 protein expression (Figure 5(f)). The AZD5363-mediated effect was similar to that of curcumin treatment with or without si-LINC00691 transfection.

## 4. Discussion

The present study revealed that LINC00691 is highly expressed in B-CPAP cells. LINC00691 inhibition suppressed proliferation and promoted apoptosis by enhancing the expression of proapoptosis-related proteins Bad and cleaved caspase-3 and suppressed the expression of the antiapoptotic protein Bcl-2 in B-CPAP cells. Curcumin treatment decreased proliferation and increased apoptosis in B-CPAP cells. In addition, curcumin inhibited LINC00691 expression in B-CPAP cells. Numerous studies have reported the anticancer effect of curcumin by regulating different mechanisms, including lncRNA regulation. Shao et al. have shown that curcumin inhibits proliferation and induces apoptosis in hepatocellular carcinoma cells by downregulating lincROR [37]. Wang et al. have suggested that curcumin enhances apoptosis and suppresses proliferation in lung cancer cells by inhibiting lncRNA UCA1 expression [38]. In the present study, our findings indicate that curcumin suppresses proliferation and enhances apoptosis in B-CPAP cells by inhibiting LINC00691. A previous study has shown that LINC00691 overexpression promotes invasion and proliferation in gastric cancer cells [39]. In contrast, Wan et al. have demonstrated that LINC00691 overexpression inhibited the migration, invasion, and proliferation of osteosarcoma cells [40]. The distinct LINC00691 functions in various cancer cells may be associated with their specific characteristics.

Tumor cells preferentially use glucose for energy via glycolysis, which produces substantial lactic acid via a phenomenon known as the “Warburg effect,” which is an essential form of metabolic reprogramming related to cancer occurrence and development [41, 42]. LDHA and HK2 are important enzymes involved in the Warburg effect. Inhibition of LDHA and HK2 expression suppresses the Warburg effect in cancer cells [43]. The Warburg effect prevents mitochondrial ATP production, thereby resisting apoptosis and promoting tumor aggressiveness [44–46]. In the present study, curcumin treatment with/without si-LINC00691 transfection enhanced ATP levels and suppressed glucose uptake, lactic acid level, and LDHA and HK2 protein expression in B-CPAP cells, indicating that curcumin might inhibit the Warburg effect by regulating LINC00691 expression in B-CPAP cells.

Akt is a downstream protein of phosphatidylinositol 3-kinase, known to participate in the regulation of numerous cellular biological processes [47]. Inhibition of Akt phosphorylation by zinc finger protein 677 suppresses proliferation, colony formation, and tumorigenic potential and induces apoptosis and cell cycle arrest in thyroid cancer cells [48]. Accumulating evidence has demonstrated the regulatory effect of curcumin on the Akt-associated pathway. Wang et al. have reported that curcumin suppressed proliferation and enhanced apoptosis in glioblastoma cells by inhibiting Akt phosphorylation [49]. In addition, curcumin was found to attenuate the epithelial-mesenchymal transition in non-small-cell lung cancer cells by inactivating the Akt-associated signaling pathway [50]. Inactivation of the Akt-related signaling pathway suppresses the Warburg effect in various cancer cells, including osteosarcoma cells [13], hepatocellular carcinoma cells [51], and breast cancer cells [52]. Our present study showed that curcumin administration with/without si-LINC00691 transfection could inhibit p-Akt activity. Akt inhibition promoted apoptosis and suppressed the Warburg effect in B-CPAP cells.

In conclusion, the present study provides evidence that curcumin promotes apoptosis and inhibits proliferation and the Warburg effect by inhibiting LINC00691 in B-CPAP cells. Moreover, the specific molecular mechanism might be mediated through the Akt signaling pathway. This study provides a theoretical basis for the treatment of PTC with curcumin. Additional *in vivo* and clinical investigations are needed to verify these findings.

## Data Availability

The data used to support the findings of this study are included within the article.
